# Cache Valley Virus in *Aedes japonicus japonicus* Mosquitoes, Appalachian Region, United States

**DOI:** 10.3201/eid2403.161275

**Published:** 2018-03

**Authors:** Fan Yang, Kevin Chan, Paul E. Marek, Philip M. Armstrong, Pengcheng Liu, Jacob E. Bova, Joshua N. Bernick, Benjamin E. McMillan, Benjamin G. Weidlich, Sally L. Paulson

**Affiliations:** Virginia Polytechnic Institute and State University, Blacksburg, Virginia, USA (F. Yang, K. Chan, P.E. Marek, P. Liu, J.E. Bova, J.N. Bernick, B.E. McMillan, B.G. Weidlich, S.L. Paulson);; The Connecticut Agricultural Experiment Station, New Haven, Connecticut, USA (P.M. Armstrong)

**Keywords:** Cache Valley virus, viruses, Aedes japonicus japonicus, mosquitoes, vector competence, vector-borne infections, zoonoses, Appalachian Region, Blacksburg, Virginia, United States

## Abstract

We detected Cache Valley virus in *Aedes japonicus*, a widely distributed invasive mosquito species, in an Appalachian forest in the United States. The forest contained abundant white-tailed deer, a major host of the mosquito and virus. Vector competence trials indicated that *Ae. j. japonicus* mosquitoes can transmit this virus in this region.

Cache Valley virus (CVV; family *Bunyaviridae*, genus *Orthobunyavirus*) is widespread throughout North and Central America and infects many species of domestic ungulates (sheep and cattle), but white-tailed deer are a likely reservoir (*1*). Although this virus has been isolated from >30 mosquito species in several genera, the principal vectors remain unknown (*2**,**3*). However, on the basis of field isolations and laboratory transmission studies, *Anopheles quadrimaculatus* and *An. punctipennis* mosquitoes probably play major roles in its transmission cycle (*1**,**4*).

CVV infection is common in sheep and causes spontaneous abortion, stillbirth, and congenital defects (*5*). The virus is neuroinvasive in humans, and there have been 3 confirmed cases and 1 death in the United States (*2**,**6*). Medical laboratories rarely test for CVV, which underestimates its true incidence and effect on human health, but serologic studies have reported high human infection rates (up to 18%) in virus-endemic areas (*7*). We report detection of CVV in the invasive mosquito *Aedes japonicus japonicus* in Blacksburg, Virginia, USA, and demonstrate that this species is a competent vector of the virus.

## The Study

**Figure 1:**
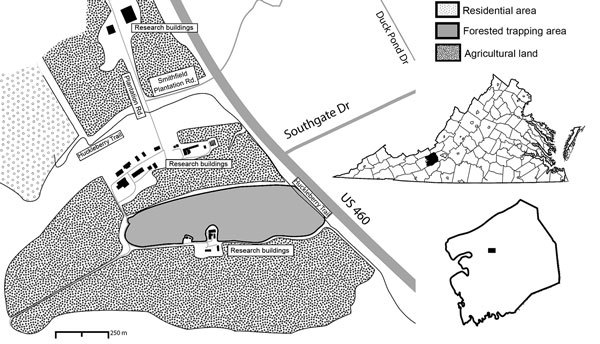
Study site used for detection of Cache Valley virus in *Aedes japonicus japonicus* mosquitoes, Blacksburg, Virginia, USA, 2015. Insets show location of Blacksburg in Montgomery County (black box) and the county in Virginia (black shading).

We collected adult mosquitoes during June 1–August 21, 2015, by using gravid traps in a forested area (area 196,115 m^2^) (Figure 1). We collected 1,197 *Ae. triseriatus* and 690 *Ae. j. japonicus* adult female mosquitoes; identified them to species on the basis of morphology; and pooled them by species, trap number, and date (Table 1). Pools (626 of *Ae. triseriatus* and 442 of *Ae. j. japonicus*) consisting of 1–50 mosquitoes were stored at −80°C. We screened samples on Vero cells for cytopathic effect and confirmed the presence of virus in positive samples by using a plaque assay (*8*).

**Table 1:** Screening of *Aedes triseriatus* and *Aedes japonicus* adult mosquitoes from gravid traps for arthropod-borne virus by using plaque assays, Appalachian Region, United States, 2015

Collection date	*Aedes triseriatus*		*Ae. japonicus*		No. positive samples
	No. mosquitoes	No. pools*	No. mosquitoes	No. pools*	
Jun 1–30	569	14	196	26	0
Jul 1–31	383	367	257	240	0
Aug 1–19	245	245	237	176	2†
Total	1,197	626	690	442	2†

*After July 21, most mosquitoes were tested individually rather than in pools. †Two Cache Valley virus–infected adult *Ae. j. japonicus* mosquitoes were collected and tested individually on August 4.

We amplified virus isolates on Vero cells to a titer of 10^5^ PFU/mL and extracted virus RNA from infected cell supernatants by using the QIAamp Viral RNA Mini Kit (QIAGEN, Valencia, CA, USA). We used reverse transcription PCR and *Bunyaviridae*-specific universal primers BCS82C and BCS332V to produce a 251-bp amplicon of the small RNA segment, which was then sequenced (*9*). Sequencing was performed by Eton Bioscience, Inc. (San Diego, CA, USA). A BLAST (http://blast.ncbi.nlm.nih.gov/Blast.cgi) query indicated that the isolates were CVV.

We amplified large RNA segments by using reverse transcription PCR and CVV-specific primers CVV_L (5′-AGTCAGCCAAAACAGCCACT-3′) and CVV_R (5′-TACAAATCTAGGGGGCATGG-3′) and amplified medium RNA segments by using primers M14C and M4510R (*9**,**10*). Resulting amplicons were sequenced and identified as CVV by performing a BLAST query. Medium RNA segments encoding the Gc protein were amplified by using the primer pair CVV_M_L (5′-CTGTCACGGTGCTAGTAGGAAAGATGTG-3′) and CVV_M_R (5′-AGTAGTGTGCTACCGGTATCAAAAACAGC-3′) and then sequenced.

We detected CVV in 2 *Ae. j. japonicus* female mosquitoes collected from different traps on August 7 (Table 1). For the week of August 4–11, we calculated the CVV minimum infection rate to be 11.5/1,000 mosquitoes (173 mosquitoes tested individually). This late-season occurrence of CVV is consistent with results of a study in Connecticut, USA (*4*). Although *Ae. triseriatus* mosquitoes were more abundant than *Ae. j. japonicus* mosquitoes and have a similar biology as the invasive mosquito, we did not detect CVV in *Ae. triseriatus* mosquitoes.

**Figure 2:**
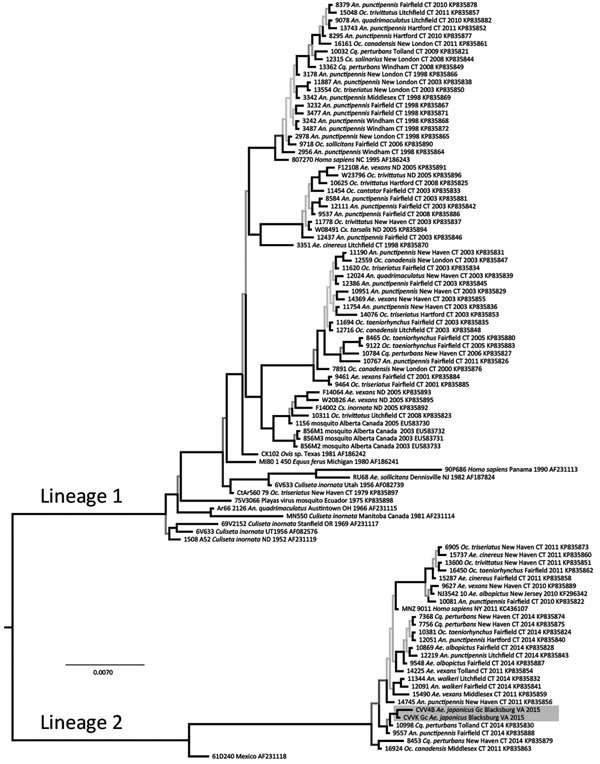
Phylogeny of Cache Valley virus (CVV) isolates in mosquitoes collected in Blacksburg, Virginia, USA (GenBank accession nos. KX583998 and KX583999), and reference isolates. The tree was inferred from the medium RNA segment of the virus polyprotein gene and estimated by using mixed model partitioned Bayesian analysis. State, year, host, and GenBank accession number are listed for each isolate. Historical lineages (1 and 2) of CVV are indicated. Shading in lineage 2 indicates strains isolated in this study. The closely related Fort Sherman virus from Panama (accession no. AF234767) is not included. Scale bar indicates expected nucleotide substitutions per site. *Ae., Aedes*; *An*., *Anopheles*; *Cq*., *Coquillettidia*; *Cx*., *Culex*; *Oc*., *Ochlerotatus*.

We used the medium RNA segment to infer phylogeny with CVV isolates reported by Armstrong et al. (*10*). We translated RNA sequences to amino acid sequences and aligned them visually, which was trivial because of invariant length and lack of insertion−deletion events. The alignment was composed of 1,803 bp from 100 isolates. We partitioned sequences by codon position and evaluated alternative models of nucleotide site substitution. We inferred phylogenetic trees by using a Markov chain Monte Carlo method in MrBayes 3.2.5 with a simultaneous estimation of topology, branch lengths, and other parameters (*11*). Stabilization of 4 concurrent chains occurred at 1 million generations, and the first 250,000 trees were discarded as a burn-in. We averaged branch lengths and other parameters and constructed a consensus tree (Figure 2) from the posterior distribution that contained support values for each clade in posterior probabilities.

CVV isolates were grouped into 2 clades or lineages (Figure 2) (*10*). Lineage 1 viruses were from the United States and Canada during 1952–2011 and lineage 2 were more recent strains from the northeastern United States. Virus isolates from Virginia were genetically similar to each other (3-bp differences) and grouped in the newly emergent lineage 2 of CVV. This monophyletic lineage shares a most recent common ancestor with a virus from Mexico isolated in 1961 (GenBank accession no. AF231118). This sister-group relationship suggests a derivation of this group from Mexico (*10*). However, additional historical samples from the region in Veracruz, Mexico, and elsewhere during the history of its introduction into the northeastern United States would further provide understanding of the phylogeography of the virus.

To determine vector competence of local mosquitoes for CVV, we established a laboratory strain from uninfected *Ae. j. japonicus* mosquitoes. Week-old female mosquitoes from the F_2_ generation were offered an infectious blood meal in a membrane feeder. This blood meal contained 1 mL of the CVV-4B isolate and 9 mL of sheep blood (Colorado Serum Company, Denver, CO, USA). We transferred postfeeding, engorged mosquitoes to 0.7-L cages and held them for 14 days at 25°C, a relative humidity of 75%, and a 16:8 (L:D) photoperiod and provided 10% sucrose. We measured rates of nondisseminated and disseminated infection (virus present in legs and wings) and oral transmission (virus present in saliva). We conducted this experiment 3 times.

Infectious blood meal titers ranged from 1.6 × 10^5^ to 4.6 × 10^6^ PFU/mL (Table 2). *Ae. j. japonicus* female mosquitoes were susceptible to oral infection with CVV and capable of transmitting the virus. After a 14-day incubation, CVV was present in 41% of abdomens, 38% of legs and wings, and 28% of saliva samples (Table 2). We found no significant differences among the 3 replicates for infection or transmission rates (p>0.05 by χ^2^ test).

**Table 2:** Rates of midgut infection, dissemination, and oral transmission of Cache Valley virus by *Aedes japonicus japonicus* mosquitoes after a 2-week extrinsic incubation, Appalachian Region, United States, 2015*

Replicate	Infectious blood meal titer, log_10_ PFU/mL	No. tested	Nondisseminated infections, %	Disseminated infections, %	Transmission, %
1	1.2 × 10^6^	18	44	39	33
2	1.6 × 10^5^	26	42	42	27
3	4.6 × 10^6^	30	37	33	27
Total	Not applicable	74	41	38	28

*Virus recovered from the midgut only was classified as a nondisseminated infection. Mosquitoes with virus-positive legs and wings were considered to have a disseminated infection. If virus was detected in salivary expectorate, it was classified as transmitting.

## Conclusions

*Ae. j. japonicus* mosquitoes are an invasive species that has spread throughout most of the eastern United States and are a competent vector of several endemic viruses (*12*). Although CVV was previously isolated from *Ae. j. japonicus* mosquitoes in the northeastern United States (*4**,**13*), we report isolation of CVV from this species in Appalachia and show that it is a competent vector of the virus. In the laboratory, vector competence of *Ae. j. japonicus* mosquitoes was equivalent to that for other species believed to be part of the CVV transmission cycle. For example, transmission rates for *An. quadrimaculatus* mosquitoes ranged from 20% to 33% after imbibing infectious blood meals with virus titers similar to those used in our study (*1*).

*Ae. j. japonicus* mosquitoes readily feed on humans and large animals, such as white-tailed deer (*12*). Consequently, this species probably contributes to local transmission of CVV. The study site is in close proximity to humans and pastured sheep and is frequented by deer (Figure 1). Therefore, all components for establishment of a focus of CVV are present. If *Ae. j. japonicus* mosquitoes are capable of transovarial transmission, as is the case with La Crosse virus, another bunyavirus (*14*), these mosquitoes could then contribute to concentrating the virus within this limited geographic area. Emergence of La Crosse virus in the Appalachian region of the United States has been associated with invasions by *Ae. j. japonicus* and *Ae. albopictus* mosquitoes (*15*). Thus, additional studies are needed to determine the role of *Ae. j. japonicus* mosquitoes in the transmission, maintenance, and presence of CVV.
